# 急性早幼粒细胞白血病中枢神经系统复发与细胞生物学特征的相关性分析

**DOI:** 10.3760/cma.j.issn.0253-2727.2021.06.014

**Published:** 2021-06

**Authors:** 世伟 杨, 荣军 马, 晓莉 袁, 丽 姜, 玉龙 李, 晓燕 董, 臻 王, 琳 张, 保军 商, 平冲 雷, 尊民 朱

**Affiliations:** 河南省人民医院血液病研究所、郑州大学人民医院血液科、河南省干细胞分化与调控重点实验室，郑州 450003 Henan Provincial People's Hospital, Institute of Hematology of Henan Provincial People's Hospital; Department of Hematology, People's Hospital of Zhengzhou University; Henan Key Laboratory of Stem Cell Differentiation and Modification, Zhengzhou 450003, China

由于三氧化二砷（As_2_O_3_）及全反式维甲酸（ATRA）在临床上的应用，急性早幼粒细胞白血病（APL）成为急性白血病中预后较好的一个亚型，但仍有5.8％的患者复发[1]。缓解后复发是除早期死亡外，影响患者预后的最主要因素，其中以中枢神经系统（CNS）复发最为常见[2]。为改善患者预后，早期识别具有CNS复发高风险的患者并积极采取防治措施具有重要临床意义，为此，我们对2013−2020年河南省人民医院血液科收治的224例持续缓解和13例CNS复发APL患者进行回顾性分析，现总结如下。

## 病例与方法

1. 一般资料：2013年1月至2020年5月河南省人民医院血液科收治初诊APL患者257例，APL诊断和疗效评估符合文献[1]标准。早期死亡16例（6.2％），复发17例（6.7％），其中CNS复发13例（5.1％），骨髓复发4例（1.6％），APL CNS复发的诊断标准：脑脊液（CSF）中原始细胞≥0.005×10^9^/L，或影像学可见颅内肿块或伴有神经系统症状和体征[2]–[3]。排除早期死亡患者，本研究共收集包括持续缓解患者224例及CNS复发患者13例共237例APL患者的资料进行回顾性分析。

2. 治疗方案：所有患者均采用双药或三药诱导治疗。三药方案：ATRA 20 mg·m^−2^·d^−1^，第1～28天，口服；亚砷酸（ATO）0.16 mg·kg^−1^·d^−1^，第1～28天，静脉滴注；去甲氧柔红霉素（IDA）8 mg·m^−2^·d^−1^，第3、5、7天，静脉滴注。双药方案：ATRA 20 mg·m^−2^·d^−1^，第1～28天，口服；ATO 0.16 m·kg^−1^·d^−1^，第1～28天，静脉滴注。巩固治疗：IDA 8 mg·m^−2^·d^−1^，第1～3天，静脉滴注；ATO 0.16 mg·m^−2^·d^−1^，第1～15天，静脉滴注，第35天使用ATRA 40 mg·m^−2^·d^−1^×20 d，口服。IDA使用4个疗程。所有患者未给予维持治疗。常规鞘内注射预防中枢神经系统白血病（CNSL），WBC<10×10^9^/L的低危患者给予甲氨蝶呤15 mg+地塞米松5 mg+阿糖胞苷50 mg×3～4次，WBC≥10×10^9^/L的高危患者给予4～6次。确诊CNS复发的患者在联合三药化疗基础上给予每周2～3次鞘内注射，脑脊液正常后每周1～2次，4～8周后，每4周1次，持续6个月[4]。疑似分化综合征患者给予地塞米松（10 mg，静脉注射，每日2次）。

3. 免疫表型及基因分析：使用美国BD公司FACSCanto流式细胞仪，检测CD34、CD56等抗体，膜抗体表达率≥20％为阳性，胞质抗体表达率≥10％为阳性[5]。采用荧光定量PCR技术检测PML-RARα、FLT3-ITD等基因[5]。

4. 随访：随访截止于2020年5月31日，中位随访时间42（0.5～88）个月。

5. 统计学处理：应用SPSS 22.0软件进行统计学分析，符合正态的计量资料采用*x*±*s*表示，两组间比较采用*t*检验；非正态分布的计量资料采用中位数（范围）表示，两组间比较采用秩和检验，两组间率的比较采用卡方检验或Fisher确切概率检验，全部统计方法均采用双侧检验，采用Kaplan-Meier法进行单因素分析，率的比较采用Log-rank检验。采用Cox回归模型Enter法进行多因素分析。*P*<0.05为差异有统计学意义。

## 结果

1. 患者的一般情况：237例患者中，男137例，女100例，初诊时中位年龄37（4～82）岁。持续缓解组224例，男128例，女96例，初诊时中位年龄37.5（4～83）岁；CNS复发组13例，男9例，女4例，复发时中位年龄32（10～62）岁。13例CNS复发患者中3例表现为头痛，2例表现为颅神经损害，1例表现为记忆力减退，1例表现为意识障碍，6例无明显临床表现（表1）。

**表1:** 13例中枢神经系统（CNS）复发的急性早幼粒细胞白血病患者的临床特征

例号	初诊年龄（岁）	性别	初诊时出血情况	复发时CNS症状	鞘注次数	初诊WBC（×10^9^/L）	初诊HGB（g/L）	初诊PLT（×10^9^/L）	CD34	CD2	CD56	FLT3-ITD	复发距初诊时间（月）	复发后生存时间（月）/转归
1	16	男	左膝关节淤血，齿龈出血，皮肤紫癜	头痛、头晕	5	39.1	69	41	–	–	+	+	7	62/存活
2	42	女	皮肤紫癜	不明显	5	23.5	64	11	–	+	–	–	10	9/死亡
3	18	男	不明显	颅神经损害	4	7.8	63	31	+	–	–	–	39	13/存活
4	37	男	皮肤紫癜	不明显	4	70.9	87	13	–	–	–	–	53	31/存活
5	60	女	皮肤紫癜、齿龈出血	颅神经损害	5	111.7	52	19	–	+	+	+	17	3/死亡
6	14	女	皮肤紫癜	头痛	4	3.7	81	25	+	–	+	–	18	0.5/死亡
7	9	男	消化道出血	不明显	6	58.8	38	43	–	–	–	–	6	5/存活
8	56	男	鼻出血	不明显	5	35.7	102	9	–	+	–	–	43	3/死亡
9	33	男	不明显	不明显	4	3.9	57	18	+	–	–	–	9	4/死亡
10	37	男	不明显	意识障碍	6	13.6	98	84	–	–	+	+	21	56/存活
11	28	女	鼻出血	不明显	4	1.9	73	19	–	+	–	–	7	17/存活
12	26	男	不明显	记忆力明显减退	5	13.6	95	77	–	–	+	–	9	2/死亡
13	30	男	消化道出血	头痛、头晕	5	11.8	87	19	–	–	–	–	29	18/存活

2. CNS复发的相关因素分析：CNS复发组初诊时中位WBC为13.6（1.9～111.7）×10^9^/L，明显高于持续缓解组的4.15（0.2～103.0）×10^9^/L，差异有统计学意义（*Z*＝−2.551，*P*＝0.011）。CNS复发组初诊时CD56表达阳性率为38.5％，持续缓解组为8.4％，差异有统计学意义（*χ*^2^＝9.063，*P*＝0.003）。两组患者的年龄、性别、初诊时PLT、WBC峰值、CD2表达、CD34表达、CD56^+^细胞占比、FLT3-ITD表达差异均无统计学意义（表2）。

**表2:** 持续缓解组与中枢神经系统（CNS）复发组一般临床特征分析

临床特征	持续缓解组（224例）	CNS复发组（13例）	*χ*^2^/*Z*值	*P*值
年龄［岁，*M*（范围）］	37.5（4～83）	30（9～60）	−1.611	0.107
性别［例（％）］			0.324	0.569
男	128（57.1）	9（69.2）		
女	96（42.9）	4（30.8）		
初诊WBC［×10^9^/L, *M*（范围）］	4.15（0.2～103.0）	13.6（1.9～111.7）	−2.551	0.011
初诊WBC［例（％）］				
<10×10^9^/L	144（64.3）	4（30.8）	4.543	0.033
≥10×10^9^/L	80（35.7）	9（69.2）		
WBC峰值［×10^9^/L, *M*（范围）］	25.93（3.20～223.24）	49.15（5.99～156.27）	−1.762	0.078
初诊PLT［×10^9^/L, *M*（范围）］	30（3～112）	19（9～84）	−0.868	0.385
CD2表达［例（％）］			0.01	0.92
阳性	57（25.4）	4（30.8）		
阴性	167（74.6）	9（69.2）		
CD34表达［例（％）］			<0.001	1.0
阳性	60（26.8）	3（23.1）		
阴性	164（73.2）	10（76.9）		
CD56表达［例（％）］			9.063	0.003
阳性	19（8.4）	5（38.5）		
阴性	205（91.5）	8（61.5）		
CD56^+^细胞占比［％, *M*（范围）］	14.54（4.39～88.09）	14.62（4.33～89.97）	−0.728	0.466
FLT3-ITD表达［例（％）］			0.054	0.816
阳性	37（16.5）	3（23.1）		
阴性	187（83.5）	10（76.9）		
诱导方案［例（％）］			0.072	0.789
三药	78（34.8）	5（38.5）		
双药	146（65.2）	8（61.5）		

单因素分析结果显示WBC≥10×10^9^/L（*χ*^2^＝7.305，*P*＝0.007）、CD56表达（*χ*^2^＝8.798，*P*＝0.003）与CNS复发相关，PLT<40×10^9^/L（*χ*^2^＝0.157，*P*＝0.692）、CD2表达（*χ*^2^＝0.198，*P*＝0.656）、CD34表达（*χ*^2^＝0.007，*P*＝0.932）及FLT3-ITD表达（*χ*^2^＝0.784，*P*＝0.376）与CNS复发无显著相关性。多变量Cox回归分析显示，初诊时WBC≥10×10^9^/L（*RR*＝5.097，*P*＝0.012）和CD56表达（*RR*＝5.606，*P*＝0.011）是CNS复发的独立危险因素，可增加APL患者CNS复发风险。

3. 预后：截至2020年5月，复发距初诊中位时间为17（6～53）个月，复发后中位随访时间为9（1～62）个月。13例CNS复发患者中7例治疗后再次缓解且存活，2例患者再次全身复发，共6例患者死亡，其中2例死于颅内出血合并感染，2例死于肺部感染，1例死于败血症，1例死于消化道出血。

## 讨论

据文献报道，APL患者CNS复发率为1.7％～12.6％[3]，[6]。在本研究中，我们分析了237例APL患者，CNS复发率为5.48％，与文献报道大致相符。本研究中的诱导方案包括双药/三药诱导方案，ATRA的使用容易导致患者处于高白细胞状态，且ATRA可促使黏附分子CD13、CD56高表达，有利于白血病细胞渗透入CNS[7]–[8]，可能增加CNS复发风险，而As_2_O_3_难以穿过血脑屏障在颅内达到有效浓度[9]，因此，ATRA和As_2_O_3_均不利于CNS复发的防治。与其他蒽环类药物相比，伊达比星的活性代谢产物可以穿透血脑屏障[10]，且伊达比星的使用也可减少ATRA引起的高白细胞状态[11]，因此早期诱导期间使用三药方案或可对CNSL有一定的防治作用，从而降低CNS复发风险，但本研究样本量有限，需扩大使用三药诱导方案样本量进一步探讨。

初诊时高白细胞状态（WBC≥10×10^9^/L）可增加APL患者CNS复发风险[3]，这与我们的研究结果一致。FLT3-ITD基因表达对于患者预后的影响仍存在争议[12]，近期有研究表明FLT3-ITD基因表达在双药诱导治疗APL患者时并不存在不良预后影响[13]–[15]，但FLT3-ITD基因表达与高白细胞状态相关[16]，且可促进黏附因子CD56的表达，从而促进白血病细胞在CNS的浸润[17]。在本研究中，并未发现FLT3-ITD基因表达对APL患者CNS复发风险的不良影响，但发现CD56表达可增加CNS复发的发生风险，日本也报道过CD56作为APL的不良预后因子，CD56阳性患者复发率明显高于CD56阴性患者（53.8％对28.9％）[18]。CD2、CD34也是APL的不良预后因子，伴有CD2或CD34表达的患者复发率明显增加（18.8％对3.3％，*P*＝0.004；16.1％对4.3％，*P*＝0.028）[5]，但本研究中我们并未发现CD2、CD34表达可增加APL患者CNS复发风险。据报道初诊时颅内出血也是CNS复发的高危因素[6]，但本研究中并未观察到CNS复发组患者初诊时有明显的颅内出血症状，仍需进一步扩大样本量进行研究。因此，对于初诊时WBC≥10×10^9^/L和（或）CD56表达的APL患者，早期采取防治CNS复发的措施，规范进行鞘内注射具有重要意义。

本研究初步显示初诊时CD2表达、CD34表达、FLT3-ITD基因表达与APL患者CNS复发无显著相关性，初诊时WBC≥10×10^9^/L和CD56表达是APL患者CNS复发的独立危险因素，可增加CNS复发风险。但本研究为单中心、回顾性分析，这些发现尚待多中心、大系列、前瞻性研究来加以确证。

## References

[1] 马 荣军, 朱 尊民, 袁 晓莉 (2017). 全反式维甲酸、砷剂联合蒽环类药物诱导方案治疗急性早幼粒细胞白血病的疗效分析[J]. 中华血液学杂志.

[2] 张 之南, 单 渊东, 李 蓉生 (2004). 协和血液病学[M].

[3] 杨 力, 胡 彩华, 董 剑明 (2013). 24例急性早幼粒细胞白血病合并中枢神经系统浸润临床分析[J]. 中华血液学杂志.

[4] 达 万明, 裴 雪涛 (2003). 现代血液病学[M].

[5] 马 荣军, 朱 尊民, 袁 晓莉 (2017). 成人急性早幼粒细胞白血病预后相关因素分析[J]. 中华血液学杂志.

[6] Montesinos P, Díaz-Mediavilla J, Debén G (2009). Central nervous system involvement at first relapse in patients with acute promyelocytic leukemia treated with all-trans retinoic acid and anthracycline monochemotherapy without intrathecal prophylaxis[J]. Haematologica.

[7] Sanz MA, Tallman MS, Lo-Coco F (2005). Practice points, consensus, and controversial issues in the management of patients with newly diagnosed acute promyelocytic leukemia[J]. Oncologist.

[8] Raanani P, Shpilberg O, Ben-Bassat I (2007). Extramedullary disease and targeted therapies for hematological malignancies--is the association real?[J]. Ann Oncol.

[9] 孙 艳花, 陈 子兴, 吴 伟林 (2009). 110例成人中枢神经系统白血病患者临床分析[J]. 中华血液学杂志.

[10] Dreyer ZE, Kadota RP, Stewart CF (2003). Phase 2 study of idarubicin in pediatric brain tumors: Pediatric Oncology Group study POG 9237[J]. Neuro Oncol.

[11] Iland HJ, Bradstock K, Supple SG (2012). All-trans-retinoic acid, idarubicin, and IV arsenic trioxide as initial therapy in acute promyelocytic leukemia (APML4)[J]. Blood.

[12] Barragán E, Montesinos P, Camos M (2011). Prognostic value of FLT3 mutations in patients with acute promyelocytic leukemia treated with all-trans retinoic acid and anthracycline monochemotherapy[J]. Haematologica.

[13] Iland HJ, Collins M, Bradstock K (2015). Use of arsenic trioxide in remission induction and consolidation therapy for acute promyelocytic leukaemia in the Australasian Leukaemia and Lymphoma Group (ALLG) APML4 study: a non-randomised phase 2 trial[J]. Lancet Haematol.

[14] Cicconi L, Divona M, Ciardi C (2016). PML-RARα kinetics and impact of FLT3-ITD mutations in newly diagnosed acute promyelocytic leukaemia treated with ATRA and ATO or ATRA and chemotherapy[J]. Leukemia.

[15] Sanz MA, Fenaux P, Tallman MS (2019). Management of acute promyelocytic leukemia: updated recommendations from an expert panel of the European Leukemia Net[J]. Blood.

[16] Gale RE, Hills R, Pizzey AR (2005). Relationship between FLT3 mutation status, biologic characteristics, and response to targeted therapy in acute promyelocytic leukemia[J]. Blood.

[17] Ferrara F, Morabito F, Martino B (2000). CD56 expression is an indicator of poor clinical outcome in patients with acute promyelocytic leukemia treated with simultaneous all-trans-retinoic acid and chemotherapy[J]. J Clin Oncol.

[18] Ono T, Takeshita A, Kishimoto Y (2014). Expression of CD56 is an unfavorable prognostic factor for acute promyelocytic leukemia with higher initial white blood cell counts[J]. Cancer Sci.

